# Comparison of Bicompartmental Knee Arthroplasty and Bicruciate-Retaining Total Knee Arthroplasty With Midterm Clinical Outcomes

**DOI:** 10.7759/cureus.90048

**Published:** 2025-08-14

**Authors:** Gai Kobayashi, Yohei Naito, Shine Tone, Masahiro Hasegawa

**Affiliations:** 1 Department of Orthopaedic Surgery, Mie University Graduate School of Medicine, Tsu, JPN

**Keywords:** bicompartmental knee arthroplasty, bicruciate retaining total knee arthroplasty, clinical outcome, patellofemoral arthroplasty, unicompartmental knee arthroplasty

## Abstract

Purpose: To compare the clinical outcomes of bicompartmental knee arthroplasty (BKA) combined with patellofemoral arthroplasty and medial unicompartmental knee arthroplasty with bicruciate-retaining total knee arthroplasty (BCR-TKA) at a mean postoperative period of five years.

Methods: A retrospective comparative study was conducted, including 16 knees in the BKA group and 39 knees in the BCR-TKA group. Demographic data and clinical outcomes, such as the 2011 Knee Society Score (2011 KSS), Forgotten Joint Score-12 (FJS-12), and knee range of motion (knee ROM), were collected and compared between the two groups.

Results: The mean follow-up period was six years for the BKA group and five years for the BCR-TKA group. Preoperative evaluations, including the 2011 KSS (except for patient expectations) and knee ROM, showed no significant differences between the groups. At the final follow-up, no statistically significant differences were observed in the 2011 KSS or FJS-12. However, knee ROM was significantly greater in the BKA group (141.9° ± 6.0° vs. 130.0° ± 10.7° for BCR-TKA, p < 0.05). No revisions were required in the BKA group, while one knee (2.6%) in the BCR-TKA group underwent revision surgery due to persistent pain.

Conclusion: BKA demonstrated comparable patient-reported outcome measures to BCR-TKA and provided significantly greater knee ROM. BKA may be a viable treatment option for knees with degeneration and symptoms limited to the medial compartment and the patellofemoral joint.

## Introduction

Total knee arthroplasty (TKA) is widely regarded as an effective treatment for knee osteoarthritis (OA). However, studies indicate that only 20-30% of patients with knee OA exhibit degeneration in all three compartments [1]. Therefore, in certain cases, TKA may replace an intact cruciate ligament or the lateral compartment. Bicompartmental knee arthroplasty (BKA) has emerged as an alternative to TKA for localized knee OA, aiming to preserve bone and maintain native kinematics [2]. BKA, when combined with patellofemoral arthroplasty (PFA) and medial unicompartmental knee arthroplasty (UKA), has shown promising medium-term results [3]. Additionally, BKA has been reported to facilitate a more normal gait speed and higher rates of return to sports compared to TKA [4]. Similarly, bicruciate-retaining TKA (BCR-TKA), which retains both the anterior cruciate ligament (ACL) and posterior cruciate ligament (PCL), has demonstrated favorable short-term outcomes [5]. BCR-TKA has also been associated with proprioception levels comparable to those of UKA [6]. Preservation of the cruciate ligaments contributes to maintaining normal knee kinematics, resulting in improved clinical outcomes and postoperative knee range of motion (ROM) [7,8]. Despite these findings, there is limited literature directly comparing the functional outcomes of BKA and TKA. Moreover, no prior studies have evaluated BKA in comparison to BCR-TKA. We hypothesize that BKA, by preserving the lateral compartment, can achieve superior restoration of knee kinematics, ROM, and functional outcomes compared to BCR-TKA. This study aimed to compare the clinical outcomes of BKA and BCR-TKA.

## Materials and methods

Patient selection

**Figure 1:**
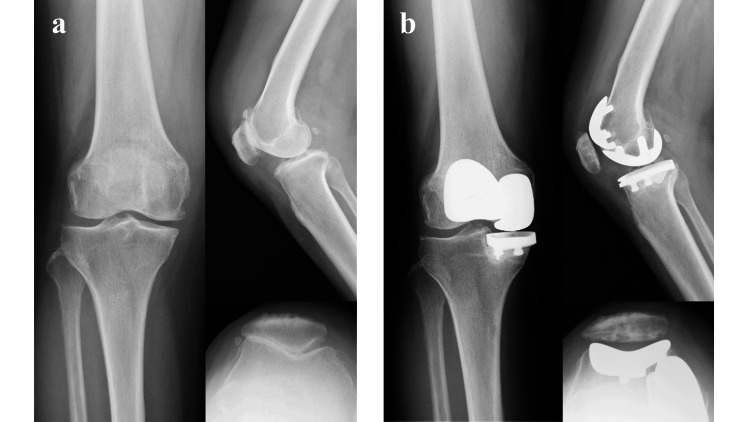
Preoperative (a) and postoperative (b) radiographs of a bicompartmental knee arthroplasty (BKA).

**Figure 2:**
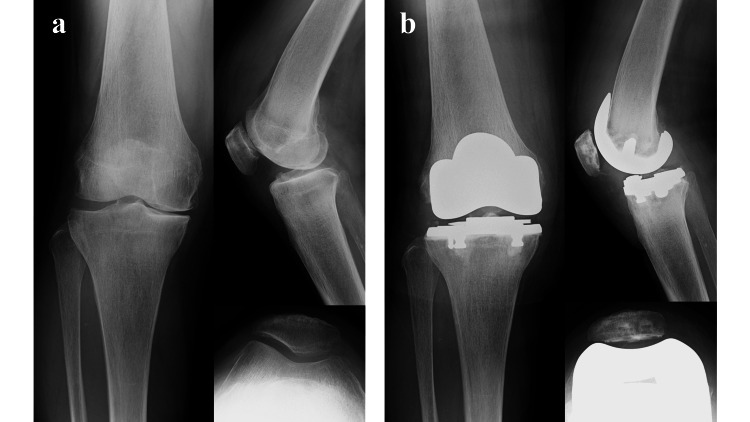
Preoperative (a) and postoperative (b) radiographs of a bicruciate retaining total knee arthroplasty (BCR-TKA).

This retrospective study included patients who underwent either primary BKA combined with PFA and medial UKA or primary BCR-TKA. In this study, we defined BKA as the combination of PFA and medial UKA. This definition is consistent with previous reports that have described this surgical approach as a type of BKA [3]. BKA procedures were performed between July 2015 and April 2017, while BKA or BCR-TKA procedures were conducted from April 2017 to October 2020 (Figures 1-2).

Among these patients, 17 knees underwent BKA, and 39 knees underwent BCR-TKA. The inclusion criteria for BKA were unicompartmental disease (medial) associated with patellofemoral OA, presenting with evident clinical symptoms, with spontaneous pain and tenderness localized to the medial compartment and the patellofemoral joint, no symptoms or signs in the lateral compartment, and intact and functional ACL and PCL. The inclusion criteria for BCR-TKA were intact and functional ACL and PCL, Kellgren-Lawrence classification Grade 3 or 4, degeneration affecting all three compartments, and patients indicated for BKA who wished to have varus deformity corrected. Retention of both cruciate ligaments in both BKA and BCR-TKA was confirmed by MRI and visualized intraoperatively. All patients in both groups were refractory to conservative treatment. The exclusion criteria for both groups were rheumatoid arthritis, previous surgery, flexion contracture >15°, and valgus deformity. The criteria were established with reference to previous studies [9-15]. One patient in the BKA group was lost to follow-up and excluded from the study. Ultimately, 16 knees in the BKA group and 39 knees in the BCR-TKA group were included in the study, with a minimum postoperative follow-up period of two years. Patient demographics, including age, gender, body mass index (BMI), and Kellgren-Lawrence classification, were recorded. All preoperative and postoperative evaluations were performed by the author.

Surgical procedures

All operations were performed by a senior surgeon (MH). At our institution, conventional TKA is generally performed for most patients with OA. However, for the present study, BKA and BCR-TKA were performed only in patients who met the inclusion criteria. PFA was conducted using the Gender Solutions PFJ system (Zimmer Biomet, Warsaw, IN). The medial UKA was performed using the following systems: ZUK (Zimmer Biomet) for nine knees, TRIBRID (Kyocera) for one knee, and the Persona Partial Knee System (Zimmer Biomet) for six knees. All BCR-TKA procedures were performed using the Vanguard XP Total Knee System (Zimmer Biomet, Warsaw, IN). The surgical technique for BCR-TKA has already been described in previous reports [9]. Preoperative computed tomography (CT) data were imported into the Zed Knee System (LEXI Co., Tokyo, Japan), a validated CT-based three-dimensional (3D) preoperative planning system for TKA. Femoral reference points were plotted on these images to define the mechanical axes of the sagittal and coronal planes, the surgical epicondylar axis (SEA), and the posterior condylar axis (PCA). Tibial reference points were also plotted to define the mechanical axis of the sagittal and coronal planes, as well as the anteroposterior axis extending from the medial third of the patellar tendon attachment to the center of the posterior cruciate ligament. For BCR-TKA, the femoral component was positioned parallel to the mechanical axis in the coronal plane and with 3° of flexion relative to the mechanical axis in the sagittal plane. In terms of rotational alignment, the femoral component was aligned parallel to the SEA in the rotational plane. If the SEA was at an angle relative to the PCA, the femoral component was positioned with 3° of external rotation relative to the PCA. In BKA, the axial rotation of the femoral component for PFA was set perpendicular to the Whiteside line. The alignment of the femoral implant in the coronal plane was optimized to ensure complete coverage of the osteotomy surface and alignment with the Whiteside line. The femoral component for medial UKA was positioned using a spacer block to achieve appropriate gap balance. In the coronal plane, the tibial component was aligned with 2° of varus in BKA, whereas, in BCR-TKA, it was positioned parallel to the mechanical axis. In both BKA and BCR-TKA, the tibial component was aligned to the patient's native posterior tibial slope in the sagittal plane. If the posterior tibial slope was ≥ 7°, the tibial component was set to 7° in the sagittal plane. For both BKA and BCR-TKA, the tibial component was aligned in the rotational plane parallel to the anteroposterior axis, defined as extending from the medial third of the patellar tendon attachment to the center of the posterior cruciate ligament. All procedures were planned using 3D-CT and executed via a mid-vastus approach. The final implant size for both BKA and BCR-TKA was determined using the gap-balancing technique. All components were cemented, and the patellae were resurfaced. The thickness of the patellectomy corresponded to the thickness of the patellar component used. Patients were permitted to walk with full weight-bearing the day after surgery, following the removal of the drainage tube.

Clinical and radiographic evaluation

**Figure 3:**
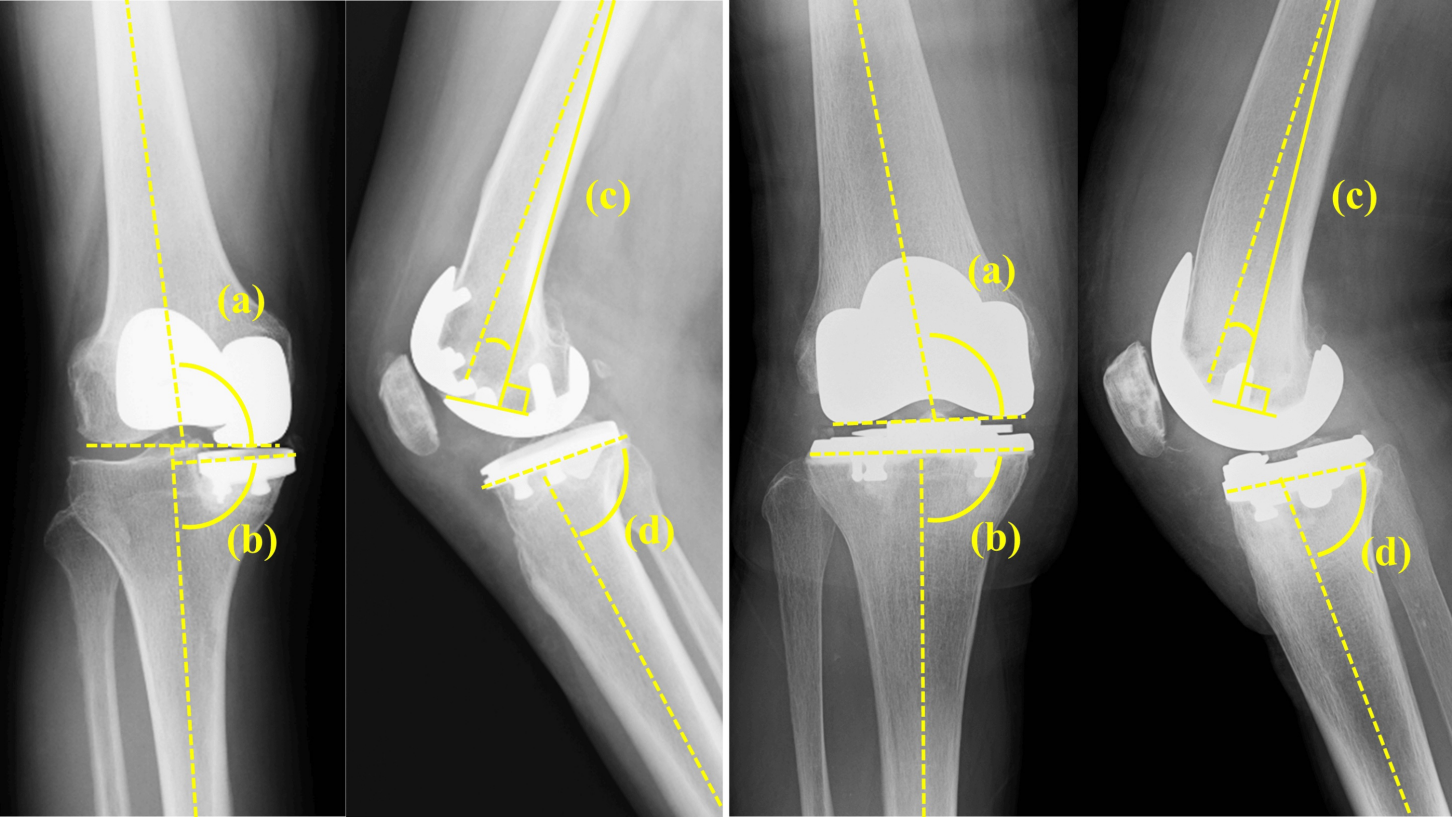
Postoperative implant installation angles. Coronal alignment is evaluated on the AP radiograph. The coronal alignment of the femoral component (a) is defined as the angle between the distal femoral component surface and the anatomic axis of the femoral shaft. The tibial component coronal alignment (b) is determined as the angle between the baseplate and the mechanical axis of the tibia. Sagittal alignment is evaluated on the lateral radiograph. The femoral component flexion angle (c) was measured as the angle between the most distal femoral fixation surface and the femoral shaft axis, with an anterior angle defined as positive. The tibial component sagittal angle (d) is determined as the angle between the baseplate and the anatomical tibial axis.

Preoperative and postoperative radiographic evaluations included full-leg standing radiographs, posterior-anterior radiographs of both knees, true lateral views, and 30° patellar axial views. The whole-leg mechanical axis was calculated on digital long-standing hip-knee-ankle radiographs by measuring the angle formed by a line connecting the center of the femoral head to the center of the knee and a second line connecting the center of the knee to the center of the talus. Radiographic outcomes related to the implants were assessed by comparing the initial postoperative radiographs with those from the last follow-up, focusing on signs of implant subsidence or loosening. Postoperative evaluation of implant installation angles included measurements of the tibial component coronal angle, tibial component sagittal angle, femoral component coronal angle, and femoral component flexion angle for UKA and BCR-TKA (Figure 3) [16,17].

Preoperative and postoperative clinical and functional evaluations included knee ROM, the 2011 Knee Society Score (2011 KSS) [18], the Forgotten Joint Score-12 (FJS-12) [19], and the revision of prostheses or replacement of any other compartment. The 2011 KSS was assessed preoperatively and at the last follow-up, while the FJS-12 was evaluated only at the last follow-up. Results from these evaluations were compared between the BKA and BCR-TKA groups.

Statistical analysis

A power analysis was conducted, determining that a minimum of 16 patients was required to achieve appropriate statistical power (β = 0.80) at a significance level of 0.05 to detect a 10-degree difference in knee ROM with a standard deviation of 10 degrees [10]. Statistical analyses were performed using EZR (Saitama Medical Center, Jichi Medical University, Saitama, Japan), a graphical user interface for R (The R Foundation for Statistical Computing, Vienna, Austria). Patient demographics were compared using the Mann-Whitney U test and Fisher's exact test. Knee ROM, 2011 KSS, and FJS-12 were analyzed using the Mann-Whitney U test. A p-value of < 0.05 was considered statistically significant.

## Results

The patient characteristics are summarized in Table 1.

**Table 1:** Patient demographics. BCR: bicruciate retaining; BMI: body mass index; BKA: bicompartmental knee arthroplasty; K-L: Kellgren-Lawrence; SD: standard deviation; TKA: total knee arthroplasty a: Mann-Whitney U test. b: Fisher's exact test. * Indicates statistical significance

Parameters	BKA	BCR-TKA	U-value	P-value
	mean ± SD	mean ± SD		
Age (years)	73.7 ± 5.9	74.7 ± 8.1	257	0.321 ^a^
Sex (Male:Female)	8:8	5:34		0.0058 ^b^*
BMI (kg/m²)	25.1 ± 3.7	24.8 ± 2.6	295	0.762 ^a^
K-L grade (3:4)	16:0	19:20		0.0001 ^b^*
Follow-up (months)	72.9 ± 18.9	62.6 ± 17.1	367.5	0.307 ^a^

No significant differences were observed between the two groups in terms of age, BMI, or follow-up period. Coronal alignment and implant installation angles are presented in Table 2.

**Table 2:** Coronal alignment and implant installation angles. BCR: bicruciate retaining; BKA: bicompartmental knee arthroplasty; SD: standard deviation; TKA: total knee arthroplasty a: Mann-Whitney U test. * Indicates statistical significance

Parameters	BKA	BCR-TKA	U-value	P-value
	mean ± SD	mean ± SD		
Pre hip-knee-ankle angle (degrees)	-5.0 ± 1.9	-8.4 ± 4.1	509	0.0003 ^a^*
Post hip-knee-ankle angle (degrees)	-2.2 ± 2.6	-0.5 ± 1.6	101	<0.0001 ^a^*
Femoral component coronal angle (degrees)	98.4 ± 2.3	96.2 ± 1.6	125	0.0005 ^a^ *
Tibial component coronal angle (degrees)	89.1 ± 0.9	89.9 ± 0.9	472	0.0011 ^a^ *
Femoral component flexion angle (degrees)	4.9 ± 3.4	3.5 ± 1.7	232.5	0.134 ^a^
Tibial component sagittal angle (degrees)	83.9 ± 3.1	82.4 ± 1.9	225.5	0.104 ^a^

Preoperative hip-knee-ankle (HKA) alignment was -5.0° ± 1.9° in the BKA group and -8.4° ± 4.1° in the BCR-TKA group, indicating significantly greater varus alignment in the BCR-TKA group. Postoperative HKA alignment was -2.2° ± 2.6° for BKA and -0.5° ± 1.6° for BCR-TKA, with the BKA group retaining significant varus alignment postoperatively. Preoperative evaluations of knee ROM and 2011 KSS, excluding the patient expectations subscale, showed no significant differences between the two groups. As shown in Table 3, both groups exhibited significant improvements in 2011 KSS and knee ROM at the final follow-up compared to preoperative values, excluding the patient expectations subscale.

**Table 3:** Comparison of preoperative outcome scores between the two groups. BCR: bicruciate retaining; BKA: bicompartmental knee arthroplasty; BMI: body mass index; K-L: Kellgren-Lawrence; KSS: Knee Society Score; ROM: range of motion; SD: standard deviation; TKA: total knee arthroplasty a: Mann-Whitney U test. * Indicates statistical significance

Parameters	BKA	BCR-TKA	U-value	P-value
	mean ± SD	mean ± SD		
2011 KSS - symptoms	9.7 ± 4.2	12.3 ± 5.3	216.5	0.0774^ a^
2011 KSS - patient satisfaction	13.6 ± 2.7	15.3 ± 5.3	223	0.097^ a^
2011 KSS - patient expectations	14.3 ± 1.1	12.5 ± 2.5	465.5	0.0035^ a^*
2011 KSS -functional activities	46.7 ± 13.8	46.9 ± 14.3	308	0.948^ a^
2011 KSS - total	84.3 ± 17.8	87.1 ± 20.6	280	0.559^ a^
Knee ROM (degrees)	118.1 ± 18.7	121.7 ± 14.7	304	0.889^ a^

A comparison of the two groups at the last follow-up is detailed in Table 4.

**Table 4:** Comparison of postoperative outcome scores between the two groups. BCR: bicruciate retaining; BKA: bicompartmental knee arthroplasty; FJS: Forgotten Joint Score; KSS: Knee Society Score; ROM: range of motion; SD: standard deviation; TKA: total knee arthroplasty a: Mann-Whitney U test. * Indicates statistical significance

Parameters	BKA	BCR-TKA	U-value	P-value
	mean ± SD	mean ± SD		
2011 KSS -symptoms	20.1 ± 3.9	21.8 ± 3.7	219.5	0.0832^ a^
2011 KSS - patient satisfaction	26.3 ± 9.7	27.8 ± 6.7	284	0.608^ a^
2011 KSS - patient expectations	9.3 ± 2.5	9.3 ± 2.3	319.5	0.889^ a^
2011 KSS -functional activities	71.2 ± 22.5	63.1 ± 20.7	387.5	0.164^ a^
2011 KSS - total	126.8 ± 36.5	121.9 ± 28.3	335	0.677^ a^
FJS-12	62.6 ± 26.3	65.7 ± 21.3	239.5	0.591^ a^
Knee ROM (degrees)	141.9 ± 6.0	130.0 ± 10.7	530	<0.0001^ a ^*

No significant differences were found in the 2011 KSS or FJS-12 between the groups. However, knee ROM was significantly greater in the BKA group. Specifically, in the BKA group, knee ROM improved from 118.1° ± 18.7° preoperatively to 141.9° ± 6.0° postoperatively at the final follow-up. In the BCR-TKA group, knee ROM improved from 121.7° ± 14.7° preoperatively to 130.0° ± 10.7° postoperatively at the final follow-up. No cases of implant loosening were observed in either group. In the BKA group, no radiographic evidence of progression of lateral compartment OA was identified, and no patients required revision surgery. In the BCR-TKA group, one knee (2.6%) underwent revision surgery due to pain one year and seven months after the initial procedure.

## Discussion

The most significant finding of this study was that the comparison between BKA and BCR-TKA, both of which preserve the ACL and PCL, showed comparable results in patient-reported outcome measures. Additionally, knee ROM was significantly greater in the BKA group. Approximately 30% of patients with knee OA experience degeneration in all three compartments [1], and studies have reported that an intact ACL is found in around 78% of patients undergoing TKA [20]. These findings suggest that for younger, physically active patients with OA limited to one or two compartments, TKA may not always be the optimal choice, and BKA should be considered as a viable alternative.

BKA offers several advantages over TKA, including minimally invasive procedures, limited bone resection, and preservation of the cruciate ligaments [2]. Additional benefits include shorter hospital stays and reduced intraoperative bleeding [21]. Several studies comparing BKA with TKA have been published. Shah et al. [22] reported that BKA and cruciate-retaining TKA (CR-TKA) produced similar functional outcomes at a two-year follow-up, with significantly greater knee ROM in the BKA group. Similarly, Tan et al. [10] reported comparable functional scores between BKA and posterior-stabilized TKA (PS-TKA) but noted greater knee ROM with BKA. Parratte et al. [23] concluded that BKA is an effective alternative to TKA, citing better ROM and FJS-12 values in BKA compared to PS-TKA. Recent studies have examined medium- and long-term outcomes. Schrednitzki et al. [11] reported comparable functional scores between BKA and CR-TKA at a five-year follow-up, with reduced blood loss and greater knee ROM in the BKA group. Goh et al. [12], in a 10-year follow-up study, observed similar clinical and functional outcomes between BKA and PS-TKA, supporting BKA as a treatment option for localized knee OA.

Tables 5-6 summarize the results of previous studies on the clinical outcomes of BKA and BCR-TKA. In this study, we hypothesized that BKA would result in better functional outcomes compared to BCR-TKA. While patient-reported outcome measures were comparable between the two groups, knee ROM was significantly greater in the BKA group.

**Table 5:** Clinical outcomes of bicompartmental knee arthroplasty combined with patellofemoral arthroplasty and medial unicompartmental knee arthroplasty. BMI: body mass index; FJS: Forgotten Joint Score; KSS: Knee Society Score; ROM: range of motion; SD: standard deviation

Authors	Number of knees	Age (years, mean ± SD)	BMI (kg/m², mean ± SD)	Follow-up (mean)	2011 KSS (mean ± SD)	FJS-12 (mean ± SD)	ROM (degrees, mean ± SD)	Revision Rates (number of revisions)
Parratte et al. [2] (2010)	69	60.2 ± 9.4	26 ± 4	11.7 years	NA	NA	Flexion 134 ± 6	40.6% (28)
Tan et al. [10] (2013)	15	52	26.0 ± 4.2	24.3 month	NA	NA	125 ± 12	0
Shah et al. [22] (2013)	16	52.1 ± 6.4	27.6 ± 4.4	2 years	NA	NA	125 ± 11	0
Parratte et al. [23] (2015)	34	61 ± 7	27.5 ± 4	3.8 years	NA	82 ± 11	Flexion 130 ± 6	0
Romagnoli et al. [3] (2018)	41	68.8 ± 8.5	28.9 ± 4.6	5.5 years	NA	NA	123.3 ± 10.7	4.8% (2)
Schrednitzki et al. [11] (2020)	40	65.3 ± 8.9	32.9 ± 6.1	5 years	NA	53 ± 23	121 ± 12	2.5% (1)
Goh et al. [12] (2020)	26	63.8 ± 8.0	27.3 ± 3.0	10 years	NA	NA	Flexion 119.5 ± 10.9	3.8% (1)
Thilak et al. [24] (2022)	6	60.8	28.9	30.5 months	NA	67.7 ± 13.0	NA	0
Baba et al. [17] (2024)	74	69	NA	6.6 years	Total 121 ± 23.4	NA	135 (120-155)	0
Present study	16	73.7 ± 5.9	25.1 ± 3.7	6.1 years	Symptoms 20.1 ± 3.9	62.6 ± 26.3	141.9 ± 6.0	0
					Satisfaction 26.3 ± 9.7			
					Expectations 9.3 ± 2.5			
					Function activities 71.2 ± 22.5			
					Total 126.8 ± 36.5			

**Table 6:** Clinical outcomes of bicruciate retaining total knee arthroplasty. BMI: body mass index; FJS: Forgotten Joint Score; KSS: Knee Society Score; ROM: range of motion; SD: standard deviation

Authors	Number of knees	Age (years, mean ± SD)	BMI (kg/m², mean ± SD)	Follow-up (mean)	2011 KSS (mean ± SD)	FJS-12 (mean ± SD)	Knee ROM (degrees, mean ± SD)	Revision Rates (number of revisions)
Baumann et al. [6] (2017)	20	66.8 ± 6.2	30	9 months	NA	NA	Flexion 115.5 ± 13.4	0
							Extension 1.2 ± 2.1	
Christensen et al. [13] (2017)	66	65 ± 7	31 ± 5	12 months	NA	NA	122 ± 8	5% (3)
Baumann et al. [25] (2018)	34	66.2 ± 7.9	30	18 months	NA	53.4 ± 26.4	NA	0
Alnachoukati et al. [5] (2018)	146	68	30	12 months	NA	NA	121 ± 9	1.4% (2)
Pelt et al. [14] (2019)	141	64	30.3	3 years	NA	NA	NA	13.5% (19)
Kono et al. [8] (2020)	17	71.2 ± 5.9	NA	18 months	Symptoms 20.9 ± 3.3	NA	Flexion 117.9 ± 13.1	NA
					Satisfaction 30.8 ± 7.4		Extension-4.1 ± 4.4	
					Expectations 10.3 ± 2.2			
					Function activities 78.6 ± 17.3			
Kalaai et al. [26] (2020)	61	65.2 ± 7.1	27.5 ± 7.1	3 years	NA	58.4 ± 33.7	NA	1.6% (1)
Kalaai et al. [27] (2021)	61	65.2 ± 7.1	27.5 ± 7.1	2 years	NA	NA	117.6	1.6% (1)
Singh et al. [15] (2023)	133	61.5 ± 9.3	31.8 ± 6.0	2.4 years	NA	55.5 ± 29.3	NA	4.5% (6)
Present study	39	74.7 ± 8.1	24.8 ± 2.6	5.2 years	Symptoms 21.8 ± 3.7	65.7 ±2 1.3	130.0 ± 10.7	2.6% (1)
					Satisfaction 27.8 ± 6.7			
					Expectations 9.3 ± 2.3			
					Function activities 63.1 ± 20.7			
					Total 121.9 ± 28.3			

Regarding implant survival, previous studies have reported higher revision rates for BKA compared to UKA and TKA [28]. Conversely, BCR-TKA has also demonstrated a high reoperation rate, with aseptic loosening, ACL impingement, and postoperative pain identified as common failure mechanisms [14,15]. In the present study, one case of revision due to pain was observed in the BCR-TKA group, while no revisions were required in the BKA group. Additionally, no radiographic evidence of implant loosening was identified in either group.

Symptomatic patellofemoral OA is generally considered a contraindication for UKA [29], and TKA is typically selected when degeneration affects two or more compartments. However, BKA may be a viable treatment option for localized knee OA, as it offers clinical postoperative outcomes comparable to TKA and provides superior knee ROM.

This study had several limitations. First, the sample size was small, highlighting the need for further investigations with larger cohorts. Second, the follow-up period was relatively short, preventing conclusions about long-term outcomes. Long-term observation is therefore necessary to validate these findings. Third, the target coronal plane alignment differed between the BKA and BCR-TKA groups, limiting the ability to analyze the impact of postoperative alignment on the results. Fourth, there is a potential for selection bias, as patients with better preoperative knee function may have been more likely to be selected for the BKA group compared to the BCR-TKA group. However, preoperative knee ROM and 2011 KSS did not show significant differences between the two groups. Fifth, some patients who were eligible for BKA were included in the BCR-TKA group. Coronal plane alignment in Japanese patients with knee OA is often associated with varus deformity, while neutral alignment is common in healthy knees [30]. Sixth, the period of surgery differed between BKA and BCR-TKA. In this study, BCR-TKA was performed for some patients who met the indications for BKA but desired alignment correction. To better establish the efficacy of BKA, future studies should include multicenter trials with larger sample sizes and extended follow-up periods.

## Conclusions

We investigated and compared the clinical outcomes of BKA, which combines PFA and medial UKA, with BCR-TKA. At a mean postoperative follow-up of five years, patient-reported outcomes were comparable between BKA and BCR-TKA, both of which preserve the anterior and posterior cruciate ligaments. Notably, the postoperative ROM was significantly greater in the BKA. These findings suggest that BKA may be a viable treatment option for knees with degeneration and symptoms limited to the medial and patellofemoral compartments.

## References

[1] Stoddart JC, Dandridge O, Garner A, Cobb J, van Arkel RJ (2021). The compartmental distribution of knee osteoarthritis - a systematic review and meta-analysis. Osteoarthritis Cartilage.

[2] Parratte S, Pauly V, Aubaniac JM, Argenson JN (2010). Survival of bicompartmental knee arthroplasty at 5 to 23 years. Clin Orthop Relat Res.

[3] Romagnoli S, Marullo M (2018). Mid-term clinical, functional, and radiographic outcomes of 105 gender-specific patellofemoral arthroplasties, with or without the association of medial unicompartmental knee arthroplasty. J Arthroplasty.

[4] Deng W, Shao H, Tang H, Tang Q, Wang Z, Yang D, Zhou Y (2023). Better PROMs and higher return-to-sport rate after modular bicompartmental knee arthroplasty than after total knee arthroplasty for medial and patellofemoral compartment osteoarthritis. Front Surg.

[5] Alnachoukati OK, Emerson RH, Diaz E, Ruchaud E, Ennin KA (2018). Modern day bicruciate-retaining total knee arthroplasty: a short-term review of 146 knees. J Arthroplasty.

[6] Baumann F, Bahadin Ö, Krutsch W, Zellner J, Nerlich M, Angele P, Tibesku CO (2017). Proprioception after bicruciate-retaining total knee arthroplasty is comparable to unicompartmental knee arthroplasty. Knee Surg Sports Traumatol Arthrosc.

[7] Halewood C, Traynor A, Bellemans J, Victor J, Amis AA (2015). Anteroposterior laxity after bicruciate-retaining total knee arthroplasty is closer to the native knee than ACL-resecting TKA: a biomechanical cadaver study. J Arthroplasty.

[8] Kono K, Inui H, Tomita T, Yamazaki T, Taketomi S, Tanaka S (2020). Bicruciate-retaining total knee arthroplasty reproduces in vivo kinematics of normal knees to a lower extent than unicompartmental knee arthroplasty. Knee Surg Sports Traumatol Arthrosc.

[9] Tone S, Hasegawa M, Naito Y, Wakabayashi H, Sudo A (2023). Association between pre- and postoperative rotational mismatches of the femorotibial components and bones in bi-cruciate retaining and posterior stabilized total knee arthroplasty. Sci Rep.

[10] Tan SM, Dutton AQ, Bea KC, Kumar VP (2013). Bicompartmental versus total knee arthroplasty for medial and patellofemoral osteoarthritis. J Orthop Surg (Hong Kong).

[11] Schrednitzki D, Beier A, Marx A, Halder AM (2020). No major functional benefit after bicompartmental knee arthroplasty compared to total knee arthroplasty at 5-year follow-up. J Arthroplasty.

[12] Goh JK, Chen JY, Yeo NE, Liow MH, Chia SL, Yeo SJ (2020). Ten year outcomes for the prospective randomised trial comparing unlinked, modular bicompartmental knee arthroplasty and total knee arthroplasty. Knee.

[13] Christensen JC, Brothers J, Stoddard GJ, Anderson MB, Pelt CE, Gililland JM, Peters CL (2017). Higher frequency of reoperation with a new bicruciate-retaining total knee arthroplasty. Clin Orthop Relat Res.

[14] Pelt CE, Sandifer PA, Gililland JM, Anderson MB, Peters CL (2019). Mean three-year survivorship of a new bicruciate-retaining total knee arthroplasty: are revisions still higher than expected?. J Arthroplasty.

[15] Singh V, Yeroushalmi D, Christensen TH, Bieganowski T, Tang A, Schwarzkopf R (2023). Early outcomes of a novel bicruciate-retaining knee system: a 2-year minimum retrospective cohort study. Arch Orthop Trauma Surg.

[16] Meneghini RM, Mont MA, Backstein DB, Bourne RB, Dennis DA, Scuderi GR (2015). Development of a modern Knee Society radiographic evaluation system and methodology for total knee arthroplasty. J Arthroplasty.

[17] Baba R, Ohkoshi Y, Maeda T (2024). The influence of patello-femoral overstuffing after modular unlinked bicompartmental knee arthroplasty (BiKA) for medial tibio-femoral and patello-femoral osteoarthritis of the knee. J Arthroplasty.

[18] Scuderi GR, Bourne RB, Noble PC, Benjamin JB, Lonner JH, Scott WN (2012). The new Knee Society knee scoring system. Clin Orthop Relat Res.

[19] Behrend H, Giesinger K, Giesinger JM, Kuster MS (2012). The "forgotten joint" as the ultimate goal in joint arthroplasty: validation of a new patient-reported outcome measure. J Arthroplasty.

[20] Johnson AJ, Howell SM, Costa CR, Mont MA (2013). The ACL in the arthritic knee: how often is it present and can preoperative tests predict its presence?. Clin Orthop Relat Res.

[21] Kooner S, Johal H, Clark M (2017). Bicompartmental knee arthroplasty vs total knee arthroplasty for the treatment of medial compartment and patellofemoral osteoarthritis. Arthroplast Today.

[22] Shah SM, Dutton AQ, Liang S, Dasde S (2013). Bicompartmental versus total knee arthroplasty for medio-patellofemoral osteoarthritis: a comparison of early clinical and functional outcomes. J Knee Surg.

[23] Parratte S, Ollivier M, Opsomer G, Lunebourg A, Argenson JN, Thienpont E (2015). Is knee function better with contemporary modular bicompartmental arthroplasty compared to total knee arthroplasty? Short-term outcomes of a prospective matched study including 68 cases. Orthop Traumatol Surg Res.

[24] Thilak J, Nagaraja Rao S, Mohan V, Babu BC (2022). Image-based robot assisted bicompartmental knee arthroplasty versus total knee arthroplasty. SICOT J.

[25] Baumann F, Krutsch W, Worlicek M (2018). Reduced joint-awareness in bicruciate-retaining total knee arthroplasty compared to cruciate-sacrificing total knee arthroplasty. Arch Orthop Trauma Surg.

[26] Kalaai S, Scholtes M, Borghans R, Boonen B, van Haaren E, Schotanus M (2020). Comparable level of joint awareness between the bi-cruciate and cruciate retaining total knee arthroplasty with patient-specific instruments: a case-controlled study. Knee Surg Sports Traumatol Arthrosc.

[27] Kalaai S, Bemelmans YF, Scholtes M, Boonen B, van Haaren EH, Schotanus MG (2021). A short-term radiological and clinical comparison between the bi-cruciate and cruciate retaining total knee arthroplasty: a retrospective case controlled study. J Clin Orthop Trauma.

[28] Agarwal AR, Cohen JS, Fuller SI, Malyavko A, Golladay G, Thakkar SC (2023). Analysis of revision rates and complication rates among patients undergoing unicompartmental and bicompartmental knee arthroplasties when compared to total knee arthroplasty. Knee.

[29] Mittal A, Meshram P, Kim WH, Kim TK (2020). Unicompartmental knee arthroplasty, an enigma, and the ten enigmas of medial UKA. J Orthop Traumatol.

[30] Kobayashi G, Hasegawa M, Yamabe Y, Tone S, Naito Y, Sudo A (2025). Classification of coronal plane alignment of arthritic and healthy knees in Japan. Journal of Joint Surgery and Research.

